# Dual-modal in vivo assessment for electrophysical and hemodynamic characteristics of cerebral edema induced by lipopolysaccharide

**DOI:** 10.1186/s12938-022-01047-x

**Published:** 2022-11-21

**Authors:** Weitao Li, Yameng Zhang, Qian Xie, Xinping Qi, Liuye Yao, Xue Ning, Zhiyu Qian

**Affiliations:** 1 Department of Biomedical Engineering, Nanjing University of Aeronautics and Astronautics, No.29 Jiangjun Avenue, Nanjing, 211106 China; 2 Department of Computer Engineering, Nanjing Institute of Technology, No.1 Hongjing Avenue, Nanjing, 211167 China; 3 School of Pharmacy, Nanjing Medical University, 101 Longmian Avenue, Nanjing, 211166 China

**Keywords:** Optical–electrical joint system, Neuro-electrophysiology, Spectrum signal, Laser speckle contrast imaging

## Abstract

The pathological features of cerebral edema are complicated. The intracranial pressure (ICP) is regarded as the most important indicator for monitoring cerebral edema. Recently, multi-parameter has been used to explore the types and pathogenesis of cerebral edema and design effective treatment strategies. This research focused on investigating the characteristic of the cerebral edema induced by lipopolysaccharide (LPS) in rats by using simultaneous electrophysical and hemodynamic parameters. The results showed that neurophysiologic parameters (firing rate (FR) and the power spectrum of local field potential (LFP power)) and hemodynamic parameters (relative concentration of oxygenated hemoglobin (ΔC_HbO2_), relative concentration of deoxyhemoglobin ΔC_HbR_) and relative cerebral blood flow (rCBF)) were linearly correlated, and the Pearson’s correlation coefficient was changed by pathological progression of cerebral edema induced by LPS. Furtherly, the treatment after two agents were observed successfully through these multi-parameters. Our findings revealed the relationship between neural activity and hemodynamic response during the progression of cerebral edema and provided a multi-parameter solution for cerebral edema functional monitoring and anti-edema drug efficacy evaluation.

## Introduction

Cerebral edema is a potentially fatal disorder which is present with many common cerebral pathologies, such as stroke [1], traumatic brain injury (TBI) [2], central nervous system tumors [3] and intracerebral hemorrhage [4]. Previous studies have been devoted on unraveling the clinical and pathological features of cerebral edema and evaluating the therapeutic effects of various drugs for cerebral edema by intracranial pressure (ICP) [5–7]. However, it is difficult to effectively distinguish the pathological characteristics of cerebral edema and examine the effects of anti-edema drugs merely based on ICP.

As the normal brain function is facilitated by a tight coupling among neuronal activity, cerebral blood flow, and metabolism [8]. The alterations of neurovascular function can be closely related to the neurological disease state and the impairment of neurovascular function was found during brain disorders including cerebral edema. Matilde found that subarachnoid hemorrhage could result in acute changes in the cerebral microcirculation [9]. Similarly, Brad et al*.* monitored that brain activity and the time-dependent neurophysiological and hemodynamic response during the cerebral ischemia and reperfusion process [10]. Damage to the BBB is one of the consequences of stroke with the greatest impact. Faraci addressed the cerebral vascular disease and neurovascular injury in stroke, and found that the damage to the blood–brain barrier is one of the major consequences of ischemia [34]. He observed a pronounced neurovascular dissociation that occurs immediately after small-scale strokes, which became the most severe a few days after and varied with the level of ischemia by multimodal method [35].

Above all, the monitoring of neuroelectrophysical and hemodynamic parameters was thus necessary and helpful for understanding the fundamental pathophysiology of edema development as well as for identifying possible therapeutic targets of cerebral edema.

In the paper, the adoption of LPS in the experiment because it could activate the expression of inflammatory factors, such as IL-Iβ, TNF-α, etc., which might contribute to the blood brain barrier (BBB) injury, neurovascular coupling dissociation and eventually caused the formation of cerebral edema [11]. Besides, BBB was the core structure for modulating the blood–brain microcirculation and neurovascular function [12]. Nevertheless, few studies have investigated the pathological characteristics of cerebral edema from the perspective of the neural activity and hemodynamics [13]. Therefore, we herein aim to elucidate the possible relationship between neuronal and hemodynamic parameters so as to bridge the complexity of brain blood barrier during the progression of cerebral edema.

At present, cerebral edema is typically classified into vasogenic edema and cytotoxic edema dependent on the pathological characteristics. Vasogenic edema is defined as extracellular accumulation of fluid resulting from disruption of the BBB, while cytotoxic edema is defined as cell swelling caused by intracellular accumulation of fluid [14]. Effective treatment is awaited based upon targeting the pathogenesis and chemical mediators involved in the specific edema condition. For instance, those agents belonging to hypertonic macromolecular fluid would fail in treatment when BBB is completely damaged, i.e., for treating vasogenic edema, and vice versa [15]. However, the direct measurement of ICP could not yield sufficient information associated with the pathophysiological mechanisms, real-time and on-site monitoring of multi-parameters, such as electrophysical and hemodynamic parameters, is thus of significance for determining the specific anti-edema therapeutics acting on the different pathology [16].

The aim of this paper is to systematically explore the neural activity and hemodynamic response during the progression of cerebral edema induced by LPS and to assess the effects of anti-edema drugs. The dual-modal system was developed to record multi-parameters, such as electrophysiological and hemodynamic parameters. Then, the assessment method can differentiated the pathological features of LPS-induced cerebral edema during the periods from neural activity and hemodynamic response, which enabled providing a potential technical solution for clinical cerebral edema pathological development monitoring and drug efficacy evaluation.

## Results

**Fig.1:**
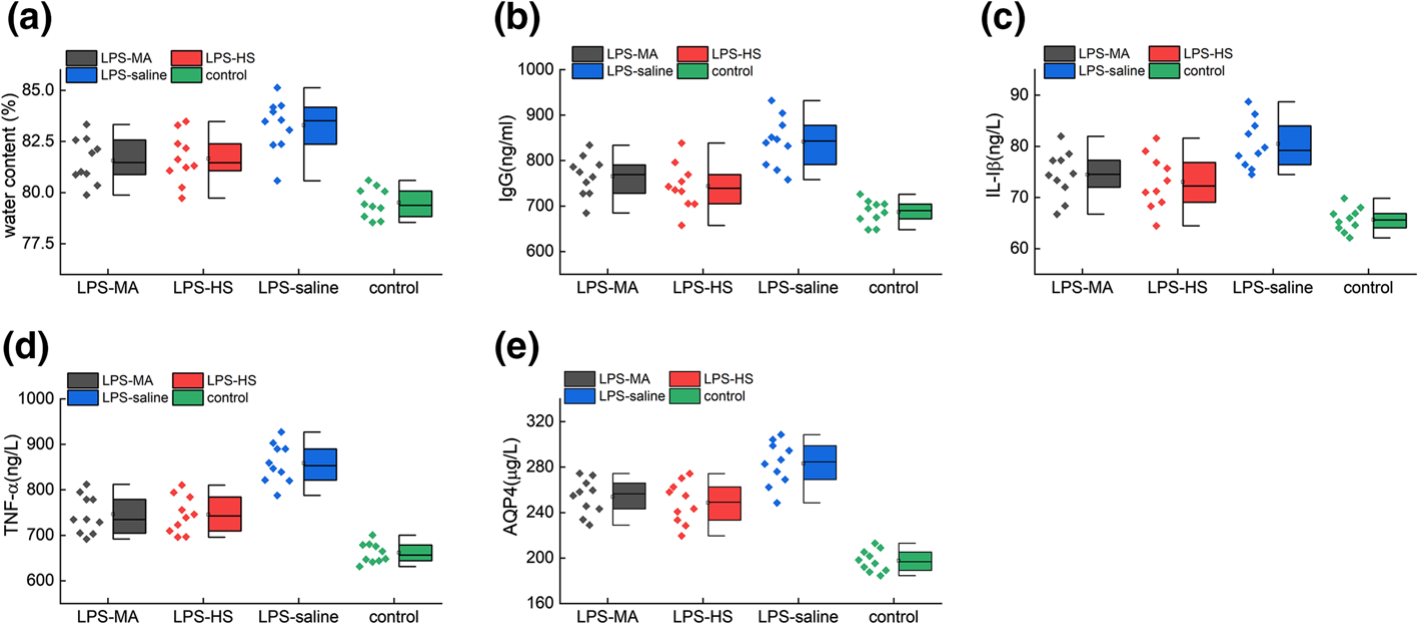
Therapeutic effects on LPS-induced cerebral edema model. Comparison of **a** cerebral water content, **b** globulin IgG, **c** inflammatory factor IL-I $\upbeta$, **d** inflammatory factor TNF-$\mathrm{\alpha }$,and **e** aquaporin AQP-4 in different treatment and control conditions, respectively

In order to validate the therapeutic effects of mannitol (MA), hypertonic saline (HS), and saline on LPS-induced cerebral edema, the brain water content (BWC) and the concentrations of multiple inflammatory factors including IL-1β, TNF-α, IgG and AQP-4 in different groups were analyzed as shown in Fig. 1 (ANOVA test, BWC: *F* = 19.634, IgG: *F* = 19.964, IL-1β: *F* = 19.096, TNF-α: *F* = 45.120, AQP-4: *F* = 48.541). The LPS-saline group rendered the highest BWC and the highest concentrations of the inflammatory factors compared with LPS-MA group (Dunnett post hoc, BWC: *P* = 0.028; IgG: P = 0.018, IL-I $\upbeta$: *P* = 0.058; TNF-$\mathrm{\alpha }$: *P*= 0.004, AQP-4: *P* = 0.009), LPS-HS group (Dunnett post hoc, BWC: *P* = 0.05; IgG: *P* = 0.004, IL-I $\upbeta$: *P* = 0.023; TNF-$\mathrm{\alpha }$: *P* = 0.002, AQP-4: *P* = 0.004) and control group (Dunnett post hoc, *P* < 0.001 for all), which indicated that LPS indeed induced the accumulation of a large amount of water and inflammatory mediators. Besides, the BWC (Dunnett post hoc: *P* = 0.001, *P* = 0.001) and concentrations of inflammatory factors in the LPS-MA group (Dunnett post hoc: *P* < 0.001 for all) and LPS-HS group (Dunnett post hoc: P < 0.01 for all) were significantly higher than those in the control group.

**Fig. 2:**
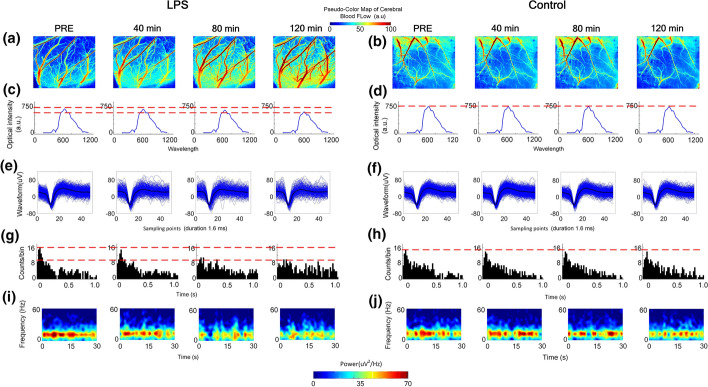
Representative rCBF in the superficial cortex in the **a** LPS-saline and **b** control group, representative IOS of the same region in the **c** LPS-saline and **d** control group, representative spikes waveforms of the ipsilateral cortex in the **e** LPS-saline and **f** control group and peristimulus time histogram (PSTH) in the **g** LPS-saline and **h** control group, representative LFPs power in the **i** LPS-saline and **j** control group, Each data was recorded in vivo pre- and post-injection, respectively

Multiple parameters were recorded to illustrate the correlations between neural activity and hemodynamic response in cerebral edema of rat. In Fig. 2, representative rCBF, original spectral and the electrophysical response of the superficial cortex from individual sample were collected by dual-modal system in vivo sequentially. Corresponding to the image of rCBF, LPS-saline group clearly displayed a noticeable velocity of rCBF increase in Fig. 2a, where that of control group remained stable in Fig. 2b. In the intrinsic optical signal (IOS) analysis, the spectral peak of LPS-saline group has dropped significantly, while that in control group kept constant in Fig. 2c, d, respectively. Furthermore, in the electrophysical signal recording, the left forepaw of the rat was stimulated using an electrical signal as synchronization reference signal. As shown in Fig. 2e–h, the counts of spikes in the cortex region were the highest at 0.06 ~ 0.08 s after the electrical stimulation, and the firing frequency in the ipsilateral cortical somatosensory region reached 16. This trend in the peristimulus time histogram (PSTH) lasted approximately 80 min after the LPS injection. Then the peak of spikes has slightly changed, the maximum counts of spikes were in the interval of 0.14 ~ 0.16 s, and the highest firing rate was reduced to 8, which showed that the exponential decline trend of PSTH gradually disappeared after 80 min. The LFPs power of the ipsilateral cortex led to a significant decrease in the power spectrum of the low-frequency band after 80 min of LPS injection in by comparing Fig. 2i, j.

**Fig. 3:**
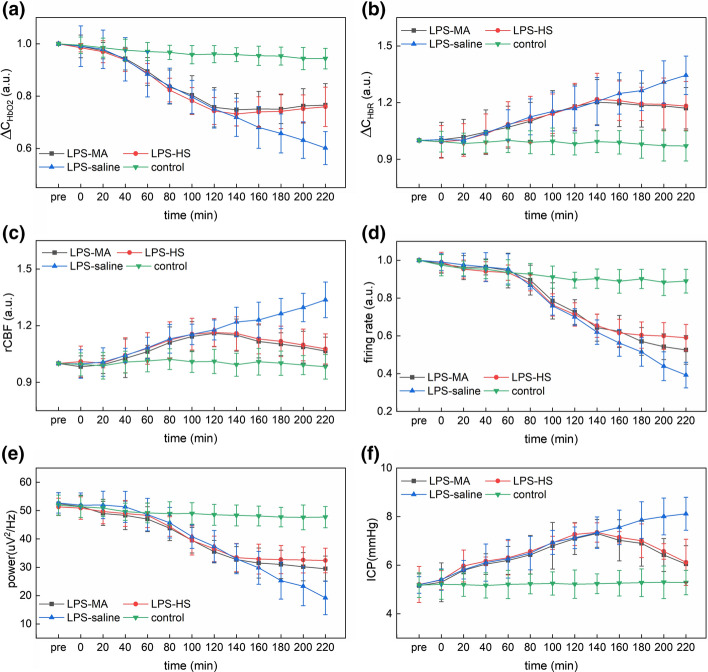
**a** Mean $\Delta {C}_{{HbO}_{2}}$, **b** mean $\Delta {C}_{HbR}$ **c** mean rCBF **d** mean firing rate, **e** mean theta LFP power and **f** mean ICP in different treatment and control conditions, respectively. All data were recorded every 20 min since the therapeutic agent was injected at the time point of 120 min. The data acquisition process lasted for 5 min at each time point

Figure 3a–c shows that the hemodynamic parameters of the LPS-saline group changed significantly from 0 to 220 min compared with control group. At 220 min, ΔC_HbO2_ was 0.602 ± 0.062 (*t* test: *P* < 0.001), ΔC_HbR_ was 1.349 ± 0.101 (*t* test: *P* < 0.001), and rCBF was 1.3364 ± 0.094 (*t* test: *P* = 0.002). In contrast to the control group, the FR and LFP of the LPS-saline group did not show significant variation within 0 to 80 min, and the FR and LFP of the PS-saline group decreased significantly within 100 to 220 min. At 220 min, the FR of the LPS-saline group was 0.402 ± 0.067 (*t* test: *P* = 0.006) and the LFP power was 20.1 ± 5.93 (*t* test: *P* = 0.008) in Fig. 3d, e. Besides, the value of all electrophysical and hemodynamic parameters in the LPS-MA and LPS-HS groups gradually approached those of the control group after injection of MA and HS treatment agents, respectively, as shown in Fig. 3a–e. The response effect of hemodynamic parameters to the therapeutic agent (MA and HS) including ΔC_HbO2_, ΔC_HbR_ and rCBF was better than that of electrophysiological parameters including FR and LFP, which was obtained by comparing the difference between the parameters of each group (LPS-MA group or LPS-HS group) and the control group within 140 to 220 min. Besides, Fig. 3f shows the average ICP value of rats, the ICP of the LPS-induced group has been rising within 0 to 120 min compared with the control group, and at 220 min ICP value of the LPS-saline was 8.12 ± 0.67 (*t* test: *P* < 0.001). When the therapeutic agent was injected into the rats, the ICP in LPS-MA and LPS-HS groups gradually decreased, and this trend was similar to that of the cortical CBF in the rats. Figure 3 suggests that the variation of the hemodynamic parameters monitored by the dual-modal system were consistent with the trend of ICP in rat induced by LPS. Notably, the trend of electrophysiological parameters was delayed to that of hemodynamic parameters and ICP, the restoration of hemodynamic parameters and recovery of neural activity had distinct time courses.

**Fig. 4:**
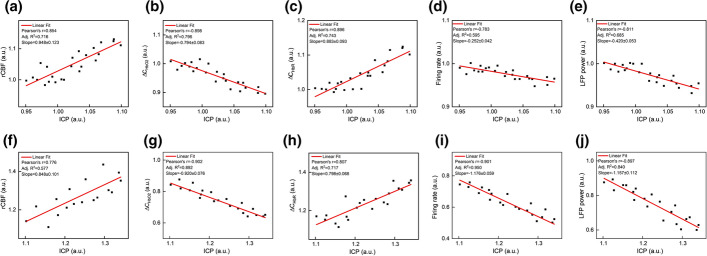
Correlation of ICP with hemodynamic and neurophysiological parameters during different time periods in the LPS-induced cerebral edema model. Pearson correlation of ICP with **a** rCBF, **b** $\Delta {\mathrm{C}}_{{\mathrm{HbO}}_{2}}$, **c**$\boldsymbol{ }\Delta {\mathrm{C}}_{\mathrm{HbR}}$, **d** firing rate and **e** LFP power after LPS injection within 0–80 min, respectively. Pearson correlation of ICP with **f** rCBF, (gb) $\Delta {\mathrm{C}}_{{\mathrm{HbO}}_{2}}$, **h** $\Delta {\mathrm{C}}_{\mathrm{HbR}}$, **i** firing rate and **j** LFP power in within 100–220 min

Furthermore, the correlations of ICP with hemodynamic and neurophysiological parameters during different time periods in LPS-saline group were analyzed as shown in Fig. 4. Figure 4a–c reveals that rCBF, ∆C_HbO2_ and ∆C_HbR_ were strongly correlated with ICP (Pearson’s *r* = 0.854, slope = 0.948; Pearson’s *r* = − 0.898, slope = -0.794; Pearson’s *r* = 0.896, slope = 0.883), while the FR and LFP power produced a weak correlation with ICP within 0–80 min (Pearson’s *r *= − 0.783, slope = − 0.252; Pearson’s *r* = − 0.685, slope = − 0.420). After 100 min, interestingly, both the Pearson’s coefficient and slope significantly increased in terms of FR (Pearson’s *r* = − 0.901, slope = − 1.176) and LFP power (Pearson’ s *R* = − 0.897, slope = − 1.157) in Fig. 4i, j, while that of rCBF (Pearson’s *r* = 0.776, slope = 0.848), ∆C_HbO2_ (Pearson’s *r* = − 0.902, slope = − 0.920)and ∆C_HbR_ (Pearson’s *r* = 0.807, slope = 0.798) had little obvious signs of change. In conclusion, the Pearson's coefficient difference FR and LPF power showed brain edema induced by LPS at about 80 min to gradually change the neuron activity.

**Fig. 5:**
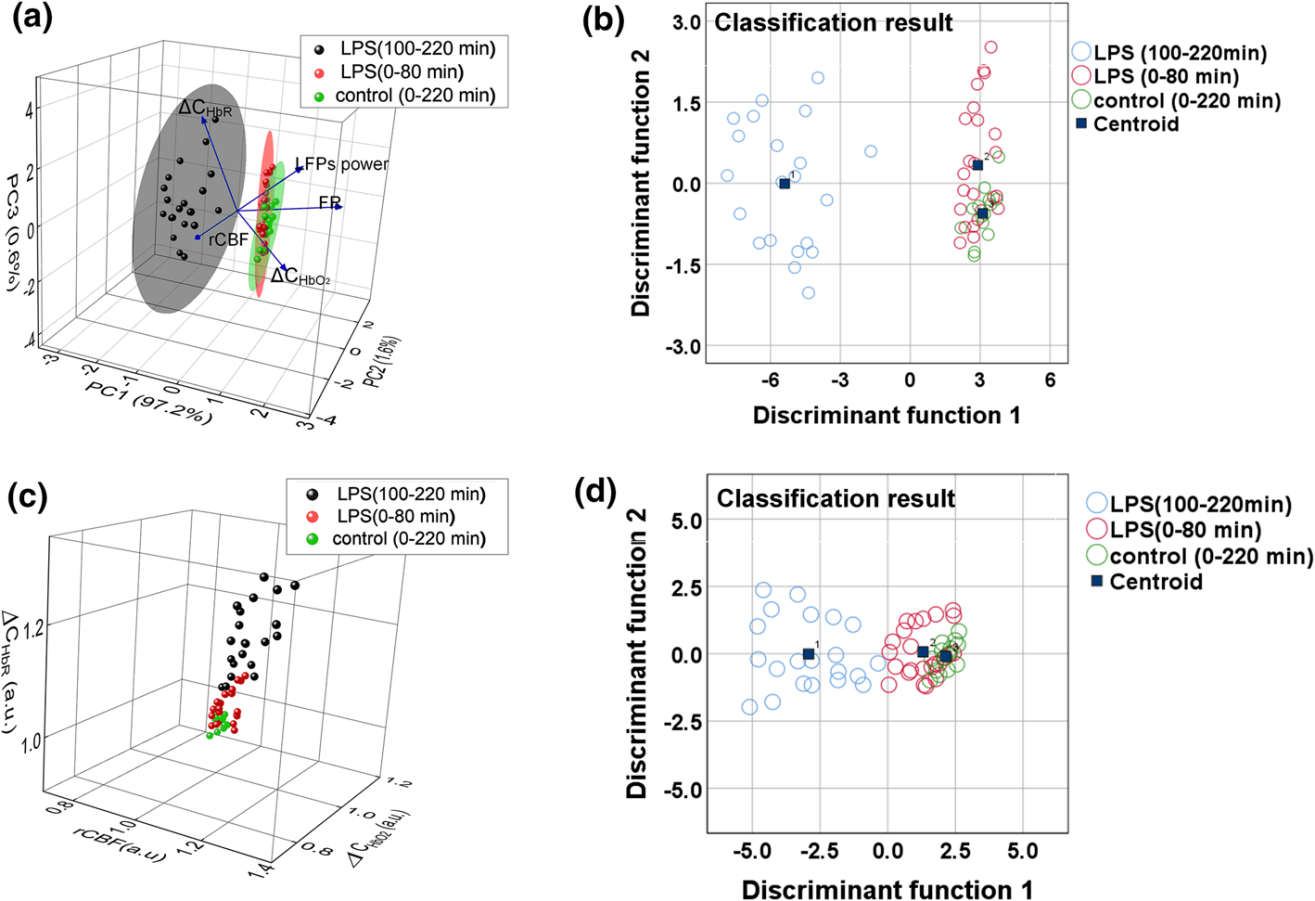
Comparison between neurovascular multi-parameter classification and vascular parameter classification. **a** Scatter diagram of principal component factor including rCBF, ∆C_HbO2_, ∆C_HbR_, firing rate and LFP power. **b** Fisher discriminant function centroid plot based on principal component factors. **c** Scatter diagram of hemodynamic parameters including rCBF, ∆C_HbO2_, and ∆C_HbR_. **d** Fisher discriminant function centroid plot based on hemodynamic parameters

Based on correlation analysis in Fig. 4, we adopted the principal component analysis (PCA) and Fisher linear discriminant to classify the pathological characteristics of cerebral edema induced by LPS within different time periods. By comparing the scatter diagrams of the PCA factors depending on rCBF, ∆C_HbO2_, ∆C_HbR_, firing rate and LFP power (Fig. 5a) or only hemodynamic parameters (Fig. 5c), it was found that the scatter diagram of the principal component factor was more distinguished by the pathological characteristics of 0–80 min and 100–220 min in LPS-saline group. Based on different parameters, we then used the Fisher linear discriminant function to classify the two types cerebral edema within 0–220 min. The correct rate of discrimination for LPS group (100–220 min) is 90.5% and it for LPS group (0–80 min) is 65.3% depending on PCA factors; however, the correct rate of discrimination for LPS group (100–220 min) is 85.7% and it for LPS group (0–80 min) is 52.1% only depending on hemodynamic parameters. Furthermore, the accuracy of the decision for control group is 78.2% depending on hemodynamic parameters, and the accuracy of the discrimination is 81.6% depending on PCA factors (the discrimination effect Wilks’ Lambda = 0.05, $\chi^{2}$ = 165.257, *P* < 0.001 and Wilks’ Lambda = 0.889, $\chi^{2}$ = 6.48. *P* = 0.039). All judgment results parameters depending on PCA factors were higher than those depending on only hemodynamic parameters. Therefore, the classification effect of the principal component factor can be clearly distinguished from pathological characteristics within 0–80 min and 100–220 min as shown in Fig. 5b and d.

**Fig. 6:**
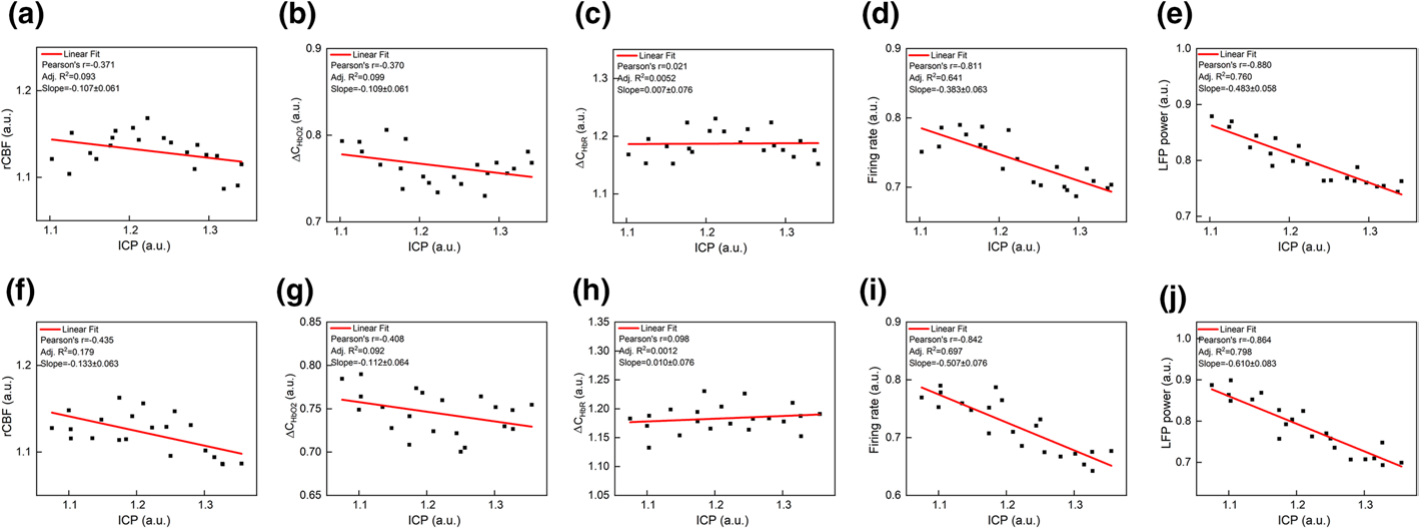
Correlation of ICP with hemodynamic and neurophysiological parameters in the treatment model. Pearson correlation of ICP with **a** rCBF, **b** ∆C_HbO2_, **c** ∆C_HbR_, **d** firing rate and **e** LFP power in LPS-HS group, respectively. Pearson correlation of ICP with **f** rCBF, **g** ∆C_HbO2_, **h** ∆C_HbR_, **i** firing rate and **j** LFP Power in LPS-MA group, respectively

We further examined the correlation of ICP with hemodynamic and neurophysiological parameters within 120–220 min in the treatment model. the Pearson’s coefficient and slope in the LPS-HS and LPS-MA groups (Fig. 6a–c and f–h) presented that ICP had weak association with hemodynamic parameters, which indicating the linear fitting degree was inappropriate for ICP and hemodynamic parameters, while the correlation of ICP with neurophysiological parameters including firing rate and LFPs power (Fig. 6d, e, i, j) approached to the state within 0–80 min in LPS-saline group. Simultaneously by studying the linear relationship between ICP and other parameters in the 2 treatment groups, the Pearson’s coefficient and slope were similar, and had no significant difference, which indicated that although the two therapeutic agents essentially (MA and HS) alleviated the degree of edema, and there is no statistical difference.

## Discussion

Neurovascular coupling, the close spatial and temporal relationship between neural activity and hemodynamics, is disrupted in pathological brain states. To understand the altered neurovascular relationship in brain disorders, simultaneous mapping of neural activity and hemodynamics is critical yet challenging to achieve. Although comparable approaches to combine optical imaging and electrophysiology in the same brain region and to simultaneously track neural and hemodynamic changes into chronic time scales are currently available. We identified a pronounced dissociation between hemodynamic and neural responses on cerebral edema induced by LPS by taking advantage of the dual-modal system to spatially tracking neurophysiological activity, rCBF, ∆C_HbO2_, and ∆C_HbR_ in the same cerebral regions relative synchronously from the pre-edema baseline to 220 min after LPS injection. The dual-modal monitoring platform was composed of the neuro-electrophysiology recording system, the IOS system, and the LSCI platform. This study explored the correlation of ICP with neural activity and hemodynamic response during the progression of LPS-induced cerebral edema. Finally, we established a discriminative model for cerebral edema induced by LPS at different time periods based on hemodynamic and neurophysiological parameters.

LPS is one of the main pathogenic components of Gram-negative bacilli, as well as a powerful inducer of inflammation [17]. For example, Wang et al. found that glial cells on striatum and cortex of rats were activated following the LPS injection, which might recapitulate the inflammatory response in cerebral hemorrhage and edema [18]. Numbers of studies have proved that LPS stimulated a variety of inflammatory factors, such as IL-Iβ, TNF-α, and IgG, caused BBB damage, and eventually contributed to cerebral edema. Here, the results in Fig. 2 verifies that LPS indeed led to an increase of ICP as well as the inflammatory factors, indicating that LPS could induce the pathological characteristics of cerebral edema of rats. The experiments of multi-parameters based on LPS-induced cerebral edema proved it feasible and researchable. Simultaneous treatment with HS and MA reduced ICP and inflammatory factors, but there was no statistical difference between the two drugs in biochemical immunoassay.

During the late stage of LPS action, LPS might reach the brain tissue through the disruptive BBB and regulate the expression of AQP-4 in glial cells, which is dominating the process of active transport of water molecules [19] and resulted in the rapid accumulation of a large amount of water and abnormal neural activity. Indeed, it is LPS-induced to pathological variation that lead to temporal uncoupling between neural activity and hemodynamics. LPS can cause endothelial cell electrical impedance to decrease in a time-dependent manner [20]. The impedance dropped in less than 1 h, indicating that at first LPS would induce the expression of inflammatory factors and lead to the destruction of BBB integrity. So CBF and SO_2_ were significantly different from those in the control group at the beginning 20 min after LPS was injected into the rat in Fig. 4a–c.

Meanwhile, since AQP-4 dominates the active quasi-transport process of water molecules, its role is to mediate the entry of water molecules from the extracellular space into the cells, which can be determined by determining the astrocytes attached to the junction of cerebrospinal fluid and blood. The AQP-4 content was used to determine the degree of cell edema. Researchers found that after 1 h, the expression of AQP-4 in glial cells under the action of LPS was significantly higher than that in the normal group, and the degree of brain edema with AQP-4 protein knockout was higher than that in the normal group [21]. Thrane et al. found that the significant expression of AQP-4 in isolated astrocytes affected by LPS was correlated with the water content of brain edema in about one hour by using two-photon imaging [22]. Therefore, this experiment results were consistent with the vitro tissue experiment [23], indicating that LPS can indeed cause abnormal AQP-4 protein in about 1 h and intracellular edema. Either of electrophysiological parameters did not produce a significant change until 80 min in the LPS-treated group in comparison with the control group in Fig. 4d and e.

In addition, the pathological progression of cerebral edema induced by LPS was complex [24], it is difficult to accurately monitor the pathological changes of LPS by relying on ICP, MRI or inflammatory factor immunoassay methods. The results in Fig. 2 and Fig. 3 showed that, hemodynamic and neuronal activity could reflect a physiologically complemental progress of LPS-induced by cerebral edema.

In the further research of the coefficient and slope within 0–120 min and 140–220 min in LPS-saline group, the results proved the disassociation between the ICP with neuronal activity. By using the principal component factor of neurovascular parameters as the characteristic classification parameters, we found that the pathological characteristics of cerebral edema induced by LPS changed obviously within 0–120 min and 140–220 min, remarkably Fisher's classification effect was better and the centroids were far away in two groups in Fig. 6. Therefore, compared with conventional ICP or blood oxygen monitoring, collaborative electrophysiological monitoring was more conducive to the in vivo study of the pathological characteristics of brain edema.

Afterwards, two therapeutic agents, MA and HS solution, were then injected into the rats through the tail vein. The results demonstrated that hemodynamic and electrophysiological parameters gradually recovered and the therapeutic agent indeed relieved the condition of cerebral edema in Fig. 3. Furthermore, the comparison of correlation coefficient and slope between ICP and neurovascular parameters pre- or post- anti-edema drug administration (time point: 120 min) suggested MA and HS therapy took a recovery effect on cerebral edema and reduced cranial osmotic pressure in Fig. 6. However, only the slope of FR and LFPs power decreased, which suggested that the treatment had little impact on neuronal discharge, that is, the therapeutic effects of both were depending on the principle of permeability, and did not substantially change the neuronal permeability caused by cytotoxicity [25].

At present, the paper only conducted immune factor experiments after the end of the experiments to verify the cerebral edema model and treatment effect. We did not deeply study the corresponding changes between inflammatory factors and hemodynamic electrophysiological parameters. In the future, it is hoped to establish the quantitative relationship between coupling parameters and the expression of intrinsic proteins in brain edema through the combination of dual-modal platform and microdialysis, and take effective treatment measures according to the difference of brain edema classification model.

## Conclusions

A dual-modal system was developed to study the multi-parameters during the progression of cerebral edema and corresponding therapeutic response in a rat model using simultaneous electrophysical and hemodynamic recording. Comprehensive hemodynamic and neurophysiological parameters, including the FR, LFPs power, rCBF, ΔC_HbO2_ and ΔC_HbR_ could be acquired and assessed concurrently to analyze pathology of cerebral edema. Our results suggested that cerebral edema induced by LPS may cause blood flow disorder and neural activity abnormity. Meanwhile, the increased ICP accelerated cerebral blood and attenuated metabolic responses, then subsequently restricted neuronal activity, which means the uncoupling between neural activity and hemodynamics during the progression of cerebral edema. We believe that the assessment method proposed in the present paper could provide a feasible technical solution for clinical edema pathological monitoring and drug efficacy evaluation.

## Materials and methods

### Animal preparation

All in vivo animal experiments were conducted in accordance with the guidelines of the Institutional Animal Care and Use Committee at Nanjing University of Aeronautics and Astronautics. The research was approved by institutional animal care and Use committee of Nanjing Medical University (IACUC-1909023). Female Sprague Dawley rats (140–180 g) from Animal Experiment Center of Nanjing Medical University (Nanjing, China) were housed in cages with food and water ad libitum. All rats were maintained in a room with a 12 h light/dark cycle and allowed a 3-day adaptation prior to experiments. The rats were randomly assigned to 4 groups, approximately 10 in each group and the experiment procedure for each group is listed in Table.1. The rats in the LPS-MA group, LPS-HS group and LPS-saline group were injected with MA, HS, and saline at 120 min after the LPS injection, respectively. Besides, the rats injected with saline during the entire process were used as control group. Afterwards the corresponding data were recorded every 20 min till 220 min for 5 min. In the experimental recording process, the rats were kept under a moderately anesthetized state, and the heart rate was at approximately 350 bpm.

**Table1:** Groups of experimental design

Groups	Dose [b.w.]	Total recording time (min)
LPS-MA	1% LPS 9 mg/kg + 20% MA 2 g/kg	220 (2nd injection, 120)
LPS-HS	1% LPS 9 mg/kg + 7.5% HS 4 ml/kg	220 (2nd injection, 120)
LPS-saline	1% LPS 9 mg/kg + 0.9% saline 1 ml/kg	220 (2nd injection, 120)
Control	0.9% saline 1 ml/kg + 0.9% saline 1 ml/kg	220 (2nd injection, 120)

### Surgical procedures

Surgical procedures were performed after animals were deeply anesthetized with 10% pentobarbital (165 mg/kg). The animal anesthesia and operation procedures were described previously in details [26]. Briefly, the cranial hole was roughly − 1.0 ~ 1.0 mm posterior to bregma and 1.0 ~ 2.0 mm left lateral to the midline for neural signal recording (A-P: − 1.0 ~ 1.0 mm, L:1.0 ~ 2.0 mm). Meanwhile, the cranial window was opened on the ipsilateral brain, and the coordinates is − 4.0 to − 1.0 mm posterior to bregma and 0 ~ 3.0 mm lateral to the midline for the IOS spectrum and laser speckle contrast imaging (LSCI) (A-P: − 4.0 ~ − 1.0 mm, L: 0 ~ 3.0 mm). Besides, a hole was buried in the right brain at a point 4 mm from the midline and 4 mm caudal to the bregma approximately (A-P: − 4.0 ~ − 3.5 mm, L: 4.0 mm) for implantation of the ICP probe.

### Dual-modal system setup and recording procedures

**Fig. 7:**
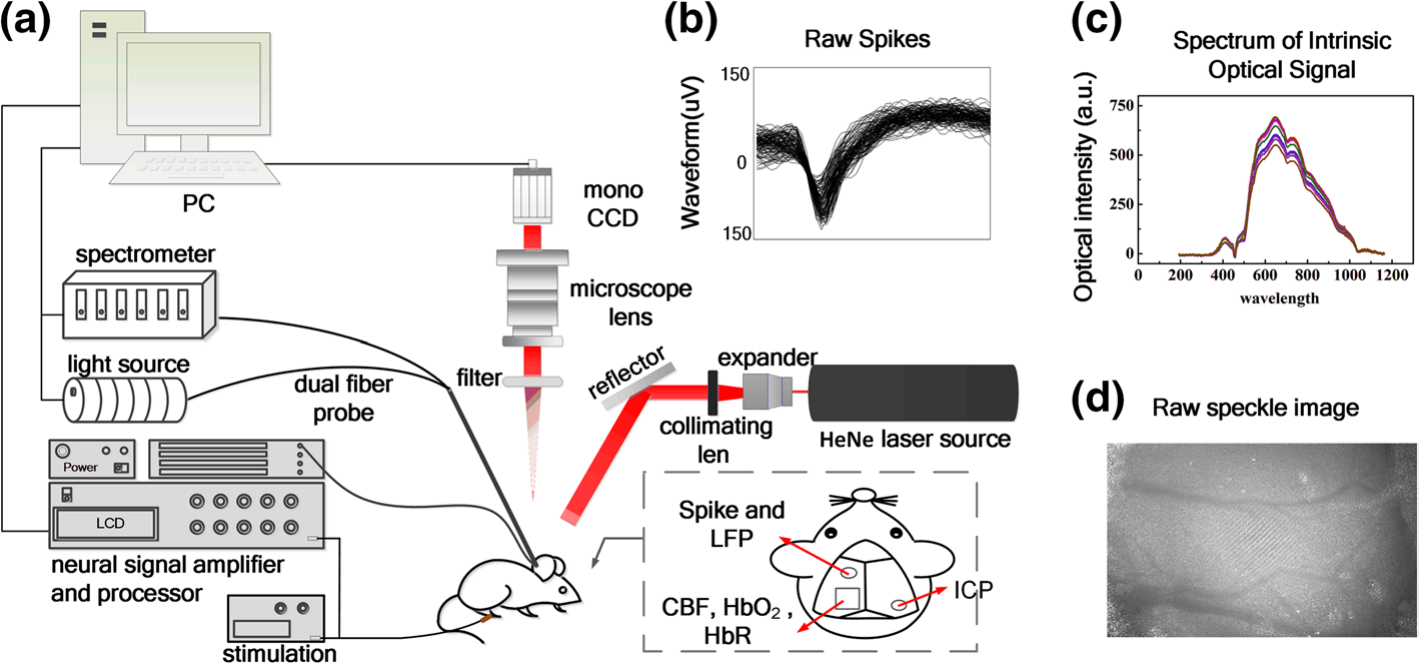
Dual-modal system for simultaneous recording of optical and neurophysiological signal. **a** Schematic diagram of the dual-modal system, **b** representative electrophysiological spikes, **c** raw spectrum of intrinsic optical signal and **d** original laser speckle contrast image of a rat

The schematic representation of our dual-modal system is shown in Fig. 7 for recording electrophysical and hemodynamic parameters, including spikes, rCBF, ΔC_HbO2_ and ΔC_HbR_. Briefly, the dual-modal system is composed of the neuro-electrophysiology recording system, the IOS spectrum acquisition system, and the LSCI platform. For avoiding the influence of laser on electrophysiological signal collection, the LSCI were obtained immediately after the electrophysiological signals were recorded for 5 min.

### Spikes and LFPs recording

The rats were implanted with a 2 × 2 microelectrode array (diameter about 33 µm, impedance more than 1 MΩ) targeting the somatosensory motor region. The arrays were inserted at a depth of approximately 600 µm below the surface of somatosensory cortex, where the largest cortical spikes and LFPs amplitudes were obtained. Four-channel LFPs and spikes were recorded simultaneously from the rat hippocampus by Cerebus Acqusition System (Blackrock Microsystems, USA). LFPs were amplified (gain: 4000), filtered (0–250 Hz) and sampled at 2000 Hz. Meanwhile, spikes (high pass filter: 0.25–5 kHz, sampled at 30 kHz) with power of more than 4.5 times the standard deviation from the baseline mean were extracted and stored with the time stamps per channel.

The detailed LFPs collection and power spectrum calculation were previously introduced in our earlier study [27]. For better comparison, an external electrical signal (5 mA) with fixed frequency (0.1 Hz) and duration (300 s) was applied by stimulating the left forepaw of the rats during the recording process as reference. The spikes were identified from the raw data and clustered by K-means method.

### *ΔC*_*HbO2*_* and ΔC*_*HbR*_* measurement by IOS*

Broadband spectrum was recorded from IOS spectrum acquisition system. A dual-fiber probe consisted of two optical fibers with the diameter of 200 m$\upmu$ and core-to-core distance of 200 m$\upmu$ was used to collect the optical spectrum. The excitation light with the wavelength from 200–1100 nm was coupled into one of the optical fibers. Then the light scattered back from the samples was collected by the other optical fiber and was detected by a spectrometer (USB 2000, Ocean Optics, USA). The obtained raw spectrum data were converted into changes of ΔC_HbO2_ and ΔC_HbR_ by the modified Beer–Lambert law [28] and least squares, seen in Eq. 1:

1 $$\Delta A=\varepsilon l\Delta C=ln({I}_{a}/{I}_{0}),$$

where *ε* is the molar absorptivity, $l$ is the path length factor, $\Delta C$ is the change in molar concentration compared to baseline measurement, ${I}_{a}$ is the post-light intensity, and ${I}_{0}$ is the baseline light intensity. Molar absorptivity and path length factor values were obtained from previous studies reported by Kuboyama et al. [29]. The concentrations of various chromophores in brain tissue, such as HbO_2_, HbR and cytochromes, can be distinguished by applying least squares to the following relationship, seen in Eq. 2 [30]:

2 $$\begin{aligned} \Delta A\left( \lambda \right) = \,& \varepsilon_{{HbO_{2} }} \left( \lambda \right)\Delta c_{{HbO_{2} }} D_{a} \left( \lambda \right) + \varepsilon_{HbR} \left( \lambda \right)\Delta c_{HbR} D_{a} \left( \lambda \right) + \varepsilon_{Cytaa3 - D} \left( \lambda \right)\Delta c_{Cytaa3 - D} D_{a} \left( \lambda \right) \\ \,\,\,\,\,\,\,\,\,\,\,\,\,\,\,\,\,\,\, & \, + \varepsilon_{Cytc - D} \left( \lambda \right)\Delta c_{Cytc - D} D_{a} \left( \lambda \right) + \varepsilon_{FAD} \left( \lambda \right)\Delta c_{FAD} D_{a} \left( \lambda \right) + \mu_{s}^{^{\prime}} \left( \lambda \right)\Delta sD_{s} (\left( { \lambda } \right). \\ \end{aligned}$$

Here, ${\varepsilon }_{Hb{O}_{2}}$, ${\varepsilon }_{HbR}$, ${\varepsilon }_{Cytaa3-D}$, ${\varepsilon }_{Cytc-D}$ and ${\varepsilon }_{FAD}$ are corresponding molar absorptivity of HbO_2_, HbR, Cytc, Cytaa3 and FAD, respectively. $\Delta {c}_{Hb{O}_{2}}$, $\Delta {c}_{HbR}$, $\Delta {c}_{Cytaa3-D}$, $\Delta {c}_{Cytc-D}$ and $\Delta {c}_{FAD}$ are corresponding changes in concentration, respectively. Given that the absorption intensity of the tissue is related to the scattering characteristics, the scattering coefficient should be regarded as another tissue chromophore. ${D}_{a}(\lambda )$ was the ratio of absorbance to absorption coefficient, ${D}_{s}(\lambda )$ was the ratio of absorbance to scattering coefficient and $\Delta s$ was the relative change of chromophore scattering. The following six wavelengths of light 450 nm, 470 nm, 500 nm, 550 nm, 570 nm and 600 nm were selected in consideration of the appropriate absorption peaks and equivalent absorption points of HbO_2_, HbR, Cytaa3-D, Cytc-D and FAD, which can be regarded as the temporal response for HbO_2_ and HbR [31].

### rCBF observation by LSCI

LSCI was employed to visualize the changes of rCBF during the entire experimental stages. In details, a He–Ne laser was adjusted by using a collimator (632.8 nm and 15 mW, Thorlabs, USA). Then the laser passed an expander and irradiated the rat brain region with a diameter of ~ 12 mm with an incident angle of 30°- 45°. The illuminated region was magnified through a zoom stereo microscope (50486A, Navitar, USA), and the raw speckle images were captured through a monochrome CCD camera (12 bit, Point Grey, Canada) with 2448 × 2048 pixels. The exposure time of the CCD was set as 20 ms, and the images were acquired through the programming software at 20 Hz. Then, a stack of 30 raw images were stored and processed. Equation 3 was used to calculate speckle contrast ($K$):

3 $$K=\frac{\delta }{<I>},$$

where $\delta$ was the standard deviation and $<I>$ was the mean of intensities within a sliding window. Here, a 5 $\times$ 5 pixels spatial window was used as the convolution window.

The CBF was calculated in Eq. 4 with the above sliding window, and the detailed algorithm was followed by our previous study and introduced earlier [32]:

4 $$CBF\propto \frac{1}{\tau }=\beta \frac{1}{T{K}^{2}},$$

where $\beta$ was a calibration parameter,$\tau is$ the speckle correlation decay time which was inversely related to $CBF$. $T$ was the exposure time of the CCD camera. For LSCI after LPS induction session, each K was then baselined against the first frame before LPS injection to calculate an estimation of relative CBF (rCBF).

### ICP measurement

To verify possible drift of the ICP, a probe (Codman & Shurtleff Inc., MA) was positioned in a water column at room temperature to obtain the pressure between 14 and 15 mmHg. Then ICP probe was inserted in the right parietal cortex and the catheter was fixed with the tape. Mean values of ICP were recorded every 20 min for 5 min and represent one measurement point in Fig. 3.

### Brain water content (BWC) measurement

At the end of the experiment, all rats were sacrificed by decapitation under the deep anesthesia state, and the brains were removed immediately. The cortical tissues (about 1 mm^3^ in volume) was extracted and placed on the electronic analytical balance to measure the wet weight (WW). The dry weight (DW) was measured after the brain tissue was dried for 24 h above 100 °C. The percent of brain water content was obtained from Eq. 5:

5 $${\text{Water content }}\left( \% \right) \, = \, \left( {{\text{WW }} - {\text{ DW}}} \right) \, \times { 1}00\% /{\text{WW}}{.}$$

### Immunohistochemistry analysis

IL-1β, TNF-α and IgG were used to evaluate the pathological characteristics of cerebral edema by ELISA kit. The detailed experimental operation was introduced earlier literature [33]. The optical density (O.D.) at 450 nm could be distinguish using a spectrometer.

### Statistics analysis

Statistical analysis was performed using SPSS software (SPSS Statistics v. 19.0, IBM Corp, Armonk, USA) by one-sample *t* test, one-way analysis of variance (ANOVA) test and repeated measures ANOVA followed by Dunnett post hoc test. Differences were considered to be statistically significant at *p* < 0.05 and *p* < 0.01. For correlation analysis, Pearson’s correlation coefficient was determined between two variables and *p* < 0.05 was considered statistically significant.

## Data Availability

The datasets used and/or analyzed during the current study are available from the corresponding author on reasonable request. All data generated or analyzed during this study are included in this published article.
